# Supplementary figures and images for: The nuclear lamina binds the EBV genome during latency and regulates viral gene expression
Source: PLoS Pathog. 2022 Apr 14;18(4):e1010400. doi: 10.1371/journal.ppat.1010400 (PMC9009669; doi:10.1371/journal.ppat.1010400)

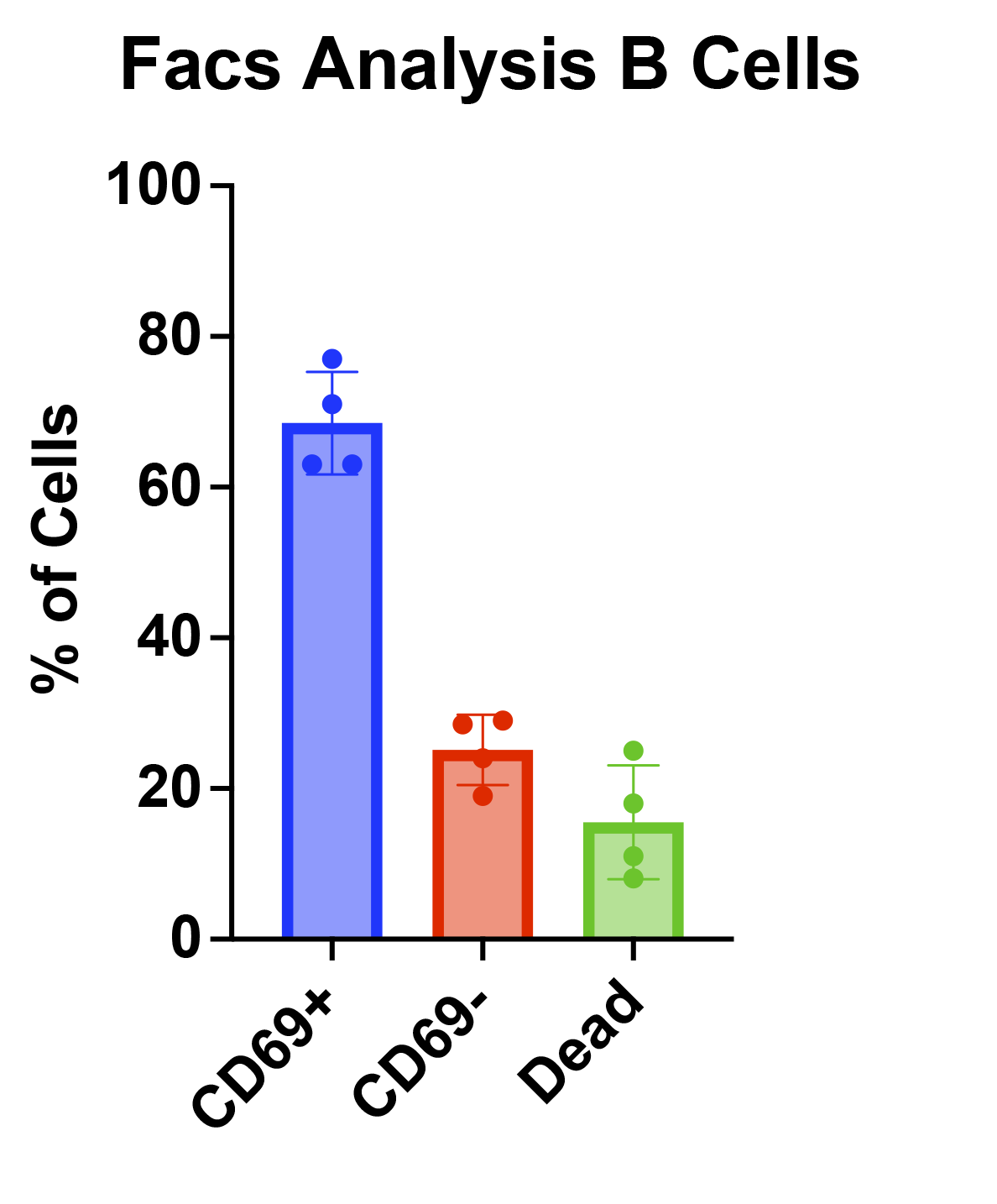

Supplement: S1 Fig — (TIF) [file ppat.1010400.s001.tif]

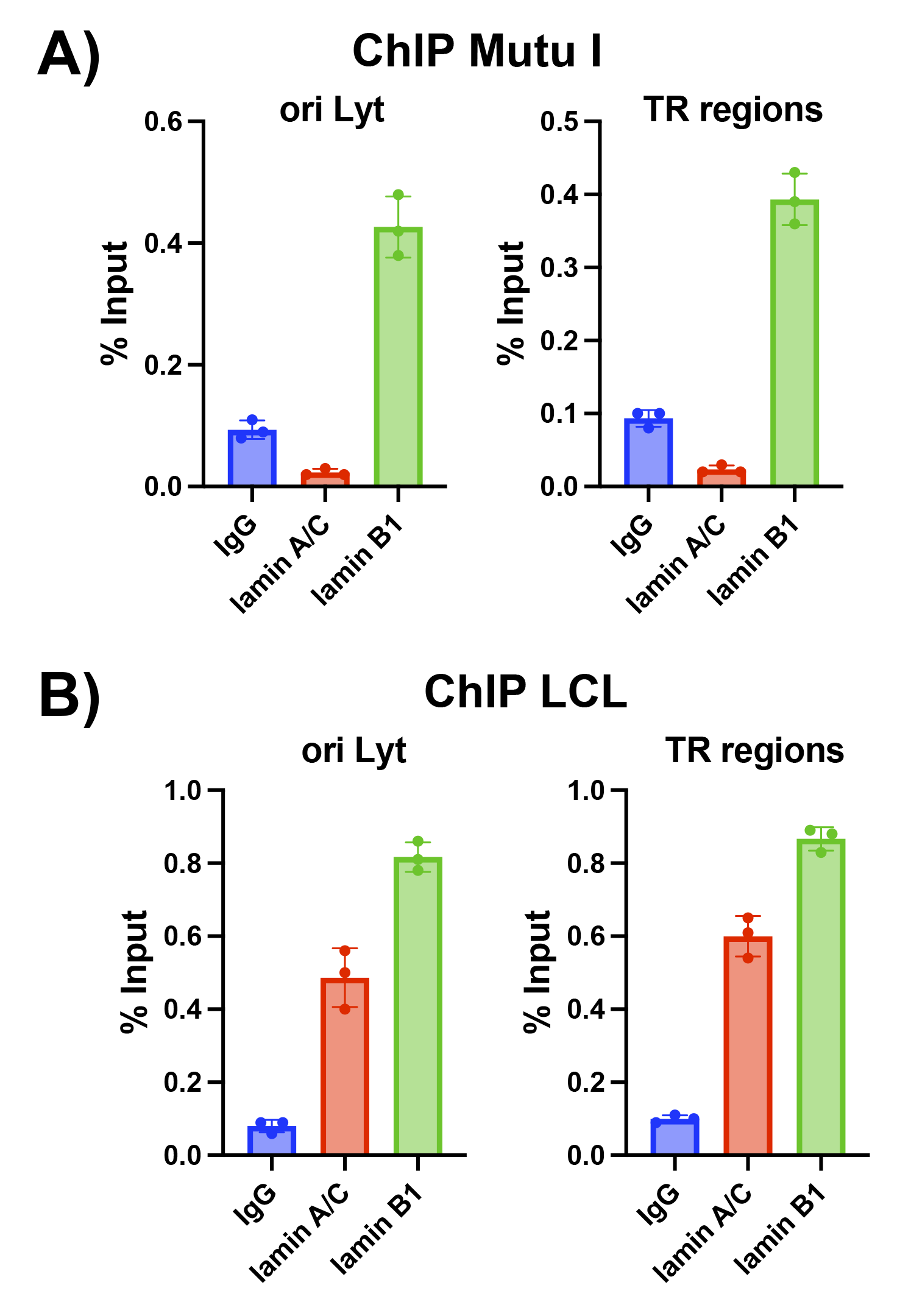

Supplement: S2 Fig — EBV chromatin was analyzed for the binding of lamin B1 and lamin A/C at the indicated EBV regions. Data are presented as %input. N = 3, Mean ± SD. (TIF) [file ppat.1010400.s002.tif]

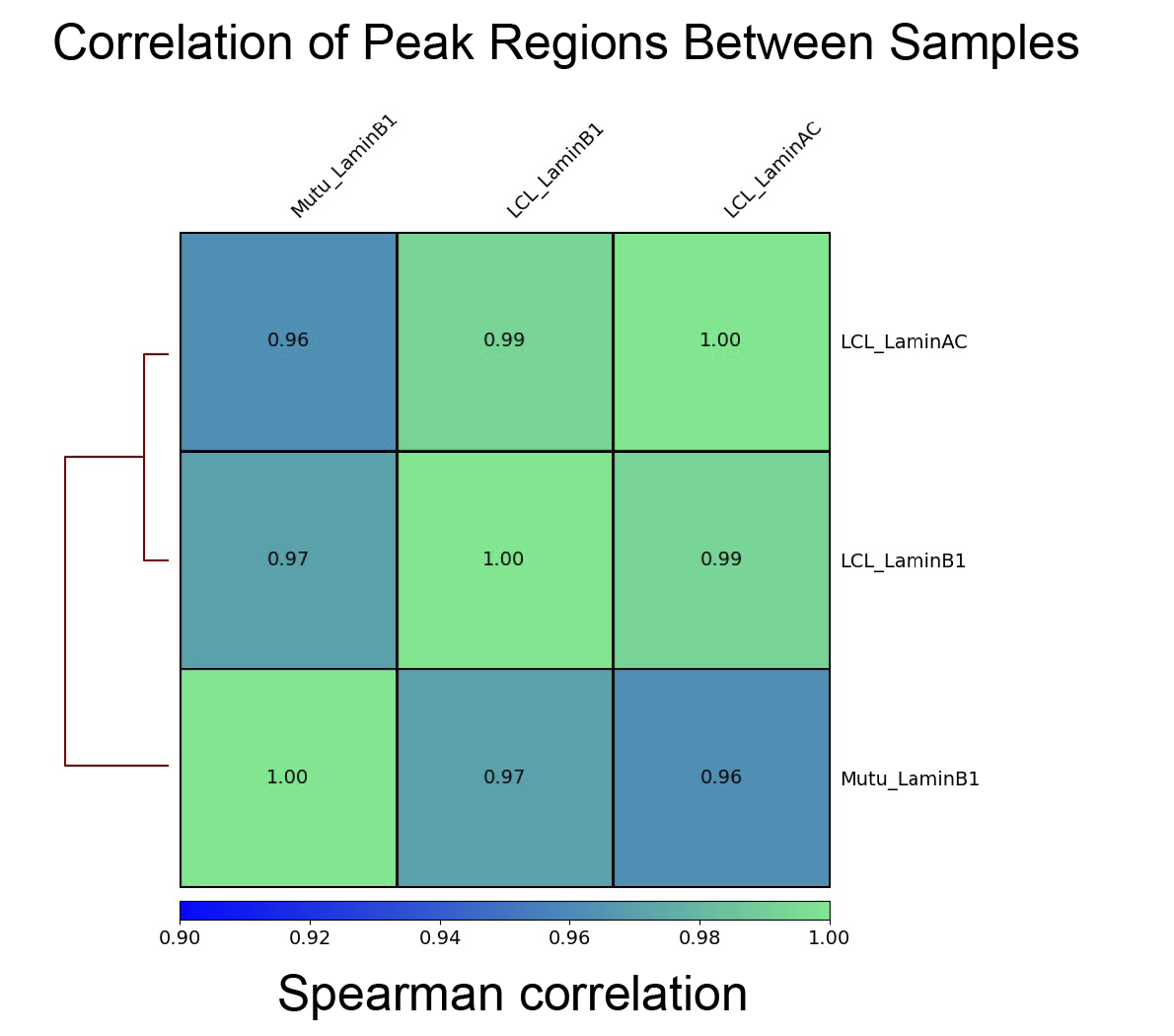

Supplement: S3 Fig — The dendrogram indicates similarity between ChIp-seq samples based on read counts. (TIF) [file ppat.1010400.s003.tif]

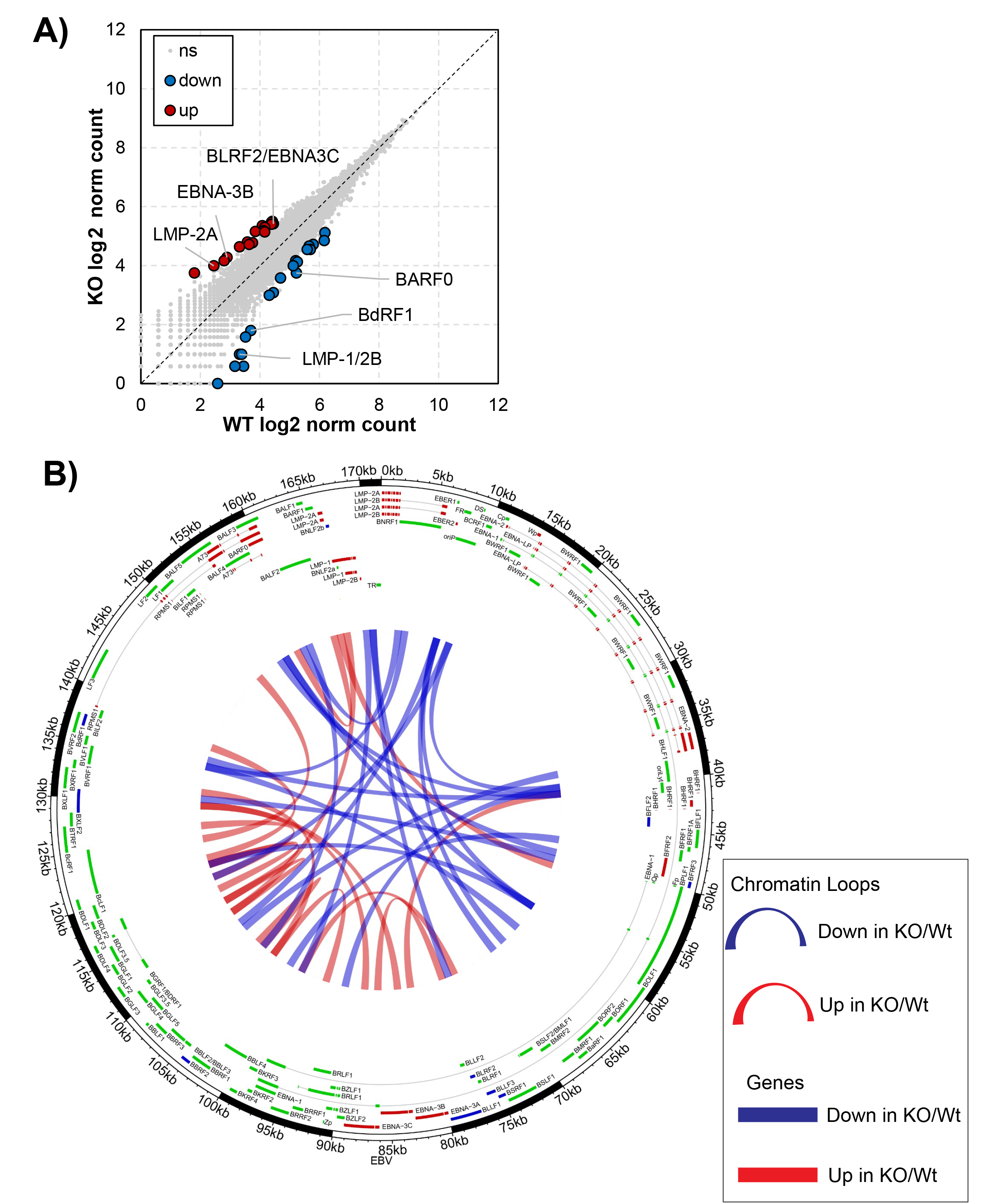

Supplement: S4 Fig — A) Scatter plots of normalized HiC counts of DNA-DNA interactions across the EBV genome in Ctr (x-axis) and LMNA KO (y-axis) LCL cells. DNA-DNA interactions with p<0.05 are indicated in blue (downregulated in Ctr cells) and red (upregulated in KO cells). B) Circos graph of all DNA-DNA contacts across the EBV genome that change between Wt and LMNA KO LCL (GM12878) cells. DNA-DNA contacts derived from HiC matrices (chromatin loops) with a p<0.05 are shown. Blue arcs represent chromatin loops that are frequent in Ctr cells; red arcs represent chromatin loops that are more frequently observed in LMNA KO cells. (TIF) [file ppat.1010400.s004.tif]
